# Supplementary material for: MKMC enables reference-free transcriptomic analysis using k-mer representations
Source: bioRxiv. 2026 Jul 10:2026.07.06.736868. Preprint. [Version 1] doi: 10.64898/2026.07.06.736868 (PMC13370388; doi:10.64898/2026.07.06.736868)
Supplement: Supplementary file 1 [file NIHPP2026.07.06.736868v1-supplement-2.pdf]

bioRxiv preprint doi: https://doi.org/10.64898/2026.07.06.736868; this version posted July 10, 2026. The copyright holder for this preprint (which was not certified by peer review) is the author/funder, who has granted bioRxiv a license to display the preprint in perpetuity. It is made available under a CC-BY 4.0 International license.

13 Supplementary Material

13.1 Details on MKMC functionalities

13.1.1 Normalization

Normalization in MKMC addresses sequencing depth and technical variability to ensure k-mer counts are comparable across samples. The toolkit offers multiple normalization methods to suit diverse datasets and analysis objectives:

- Frequency Count Normalization: Converts raw k-mer counts into relative frequencies for straightforward comparisons.
- Quantile Normalization: Aligns the distributions of k-mer abundances across samples, reducing systematic biases.
- DESeq2-like Normalization: Adapts a size factor approach for k-mer abundance matrices to account for differences in sequencing depth while controlling for outliers.

13.1.2 Dimensionality Reduction

High-dimensional k-mer datasets can be challenging to interpret and computationally expensive to analyze. MKMC provides built-in dimensionality reduction techniques to address these challenges:

- Principal Component Analysis (PCA): Projects k-mer data into orthogonal components that capture the most variation, simplifying exploratory data analysis.
- Uniform Manifold Approximation and Projection (UMAP): Preserves both local and global structure in a low-dimensional space, aiding in visualization and cluster identification.

13.1.3 Statistical Correlations

MKMC includes a comprehensive suite of tools for statistical correlation analysis, enabling users to identify associations between k-mer abundances and experimental conditions. Supported methods include:

- Pearson Correlation: Measures linear relationships between k-mer counts and continuous variables.
- Spearman Rank Correlation: Detects monotonic relationships, including non-linear patterns.
- Kendall's Tau: A robust method for analyzing ordinal data or datasets with tied ranks.

13.1.4 Two Condition Differential K-mer Discovery

K-mers are selected according to a binary conditions (control vs. disease).

T-Test:

Firstly a $\log 2(x + 1)$ transformation is applied to sample counts. Then each feature association between sample counts and labels is evaluated by p-value t-test, adjusted

bioRxiv preprint doi: https://doi.org/10.64898/2026.07.06.736868; this version posted July 10, 2026. The copyright holder for this preprint (which was not certified by peer review) is the author/funder, who has granted bioRxiv a license to display the preprint in perpetuity. It is made available under a CC-BY 4.0 International license.

by Bonferroni, Benjamini-Hochberg, Benjamini-Yekutieli or Holm-Bonferroni correction procedure for controlling false discovery rate. The top k-mers are selected from the lowest value to the highest p-values.

Signal-to-Noise ratio:

The signal to noise ratio (SNR) is a measure used to compare the level of a desired signal to the level of background noise [45]. The signal is the expression levels of k-mers, while the noise is the variability or errors in the measurements. The SNR is calculated by dividing the difference between group means by the sum of group standard deviation. The top k-mers are selected with absolute values from highest to lowest.

$$snr = \frac{|mean_C - mean_D|}{SD_C + SD_D} \tag{1}$$

Wilcoxon-rank sum test:

The normalized k-mer counts are used to conduct the Wilcoxon rank-sum test. Then each feature association between sample counts and groups is evaluated by p-value, adjusted by Bonferroni, Benjamini-Hochberg, Benjamini-Yekutieli or Holm-Bonferroni correction procedure for controlling false discovery rate. The top k-mers are selected from the lowest value to the highest adjusted p-values.

Detection of Imbalanced Differential Signal:

To detect k-mers with abnormal signal in a multi-condition experiment [46]. For each k-mer, we calculate the maximum expression in the control group.

Let the k-mer expression values of the n_1 control samples for a k-mer be given by:

$$[x_{1i}]_{i \in 1,2,\ldots,n_1}$$

and the k-mer expression values of the n_2 disease samples for a k-mer be given by:

$$[x_{2i}]_{i \in 1,2,\ldots,n_2}$$

To detect k-mers that show higher expression in control samples compared with disease samples, the maximal k-mer expression value of a k-mer among control samples as:

$$\hat{x}_1 = \max_i X_{1i}$$

The DIDS score is then given by:

$$J_{up} = \sum_{i=1}^{n_2} f(|x_{2i} - \hat{x}_1|^+)$$

where:

$$|x|^+ = \begin{cases} x & \text{if } x \geq 0, \\ 0 & \text{if } x < 0 \end{cases}$$

where f is a strictly increasing function. We use the following three variants of f:

$$f_{tanh}(x) = 1 + \tanh((3x - 3))$$
$$f_{quad}(x) = x^2$$
$$f_{sqrt}(x) = \sqrt{x}$$

bioRxiv preprint doi: https://doi.org/10.64898/2026.07.06.736868; this version posted July 10, 2026. The copyright holder for this preprint (which was not certified by peer review) is the author/funder, who has granted bioRxiv a license to display the preprint in perpetuity. It is made available under a CC-BY 4.0 International license.

13.1.5 Multiple Condition Differential K-mer Discovery

ANOVA:

The normalized k-mer counts are subjected to the ANOVA test. This statistical test assesses whether there are significant differences in mean expression levels across multiple groups. Each k-mer feature's association with group differences is evaluated using ANOVA, generating p-values indicative of significance. P-values are adjusted using Bonferroni, Benjamini-Hochberg, Benjamini-Yekutieli or Holm-Bonferroni correction procedure. Subsequently, k-mers are prioritized based on their adjusted p-values.

Detection of Imbalanced Differential Signal:

To detect k-mers with abnormal signal in a multi-condition experiment. For each k-mer, we calculate the maximum expression in the control group.

Let the k-mer expression values of the n_1 control samples for a k-mer be given by:

$$[x_{1i}]_{i \in 1,2,\ldots,n_1}$$

and the k-mer expression values of the n_2 disease samples for a k-mer be given by:

$$[x_{2i}]_{i \in 1,2,\ldots,n_2}$$

This multiple-condition filtering method returns the maximal value by iteratively estimating the DIDS score by comparing one condition against the other.

$$S_l = \max_i X_{1i}$$

13.1.6 Entropy

We add a +1 to all the counts of the k-mer matrix to avoid flow errors when taking the logarithm [37]. We calculate the entropy of a k-mer across samples using Shannon's formula. For a given k-mer k and it's counts in the different samples $S_k = (C_{k0}, C_{k1}, ..., C_{kn})$, we compute it's entropy value H_k as follows:

$$H_k = -\sum_{i=0}^{n} f_{ki} \log_2(f_{ki}) \tag{2}$$

where:

$$f_{ki} = \frac{C_{ki}}{\sum_{j=0}^{n} C_{kj}}$$

f = frequency, n is the number of samples. The top k-mers are selected from smallest to largest score.

13.2 MKMC workflow details

k-mers counting process may be parameterised in the aim of deciding which k-mers should be processed in the next stages, e.g. for excluding too rare k-mers. It is performed simply by passing proper parameters to KMC.

k-mers merging (ii) is performed independently for each KMC bin. The input is s bins, respectively for every sample, containing counts of lexicographically ordered k-mers. To merge all k-mers across samples for a given bin a binary heap is employed. The

bioRxiv preprint doi: https://doi.org/10.64898/2026.07.06.736868; this version posted July 10, 2026. The copyright holder for this preprint (which was not certified by peer review) is the author/funder, who has granted bioRxiv a license to display the preprint in perpetuity. It is made available under a CC-BY 4.0 International license.

heap is initialized with the first k-mers from every sample. The lexographically lowest sequence is on the top of the heap, and it is (if fulfills filtering criteria) the first output. After extracting the top k-mer from the heap, the next one of the corresponding sample is added to the heap, unless there are no more k-mers in the sample. Then, the same k-mer, if any, of other samples is extracted from the heap until the new k-mer is found. The single row of a matrix is created with counts of the extracted k-mers and zeros for samples that were not present in the heap, meaning that these k-mers were absent in such samples. New k-mers of the samples are constantly inserted after every extraction. The same process is repeated for the next k-mers, until all k-mers have been processed.

The rows are saved if they fulfill the given requirements of the fraction of samples with the specified k-mer counts. Keeping only k-mers belonging to the given additional set is performed by simple k-mer counting of sequences in the set. The obtained KMC file is linearly iterated while merging consecutive k-mers.

The stage is also responsible for data collection for normalization, e.g. for frequency count, it collects sums of the samples counts to divide the counts by the sum later. The data is stored in temporary binary STATS files.

Depending on the work scenario, MKMC performs a few passes of the input data or its modified form. Generally, every of the abovementioned stages perform a single pass, except correction (*iv*), which performs two passes: correction itself and storing the results.

13.3 Details on MKMC performance

We observed the time of various main MKMC stages depending on k-mer length and a number of threads (supplementary Fig. 9). The results include sequential dimensionality reduction resulting in long postprocessing.

We observed CPU utilization (32 threads) and memory usage for performing all the tasks in a single run, except dimensionality reduction (supplementary Fig. 10; similarly to Fig. 3g).

13.4 MKMC performance benchmarking experiments running

In the experiments whose results was shown on Fig. 3 and supplementary Fig. 9, 10, we used the datasets given in Table 5 and killfish (for GSE216369) genome stated in the article. We excluded the following samples from GSE132040 due to a lack of a phenotype required in some experiments. SRR9126736, SRR9126839, SRR9126882, SRR9127209, SRR9127374, SRR9127440, SRR9127538, SRR9126793, SRR9126847, SRR9126981, SRR9127233, SRR9127413, SRR9127473, SRR9127564.

Versions of the utilized tools were as follows:

- k-mer processing tools: MKMC — 1.0.0; kmtricks — 1.4.0; Kamrat — 1.2.0;
- mappers: BWA — 0.7.18; STAR — 2.7.11b;
- supporting tools: Jellyfish — 2.3.1; joinCounts has no version; featureCounts — 2.0.8; TrimGalore — 0.6.10;
- system-wide tools: apptainer — 1.4.5; Python — 3.13.9.

bioRxiv preprint doi: https://doi.org/10.64898/2026.07.06.736868; this version posted July 10, 2026. The copyright holder for this preprint (which was not certified by peer review) is the author/funder, who has granted bioRxiv a license to display the preprint in perpetuity. It is made available under a CC-BY 4.0 International license.

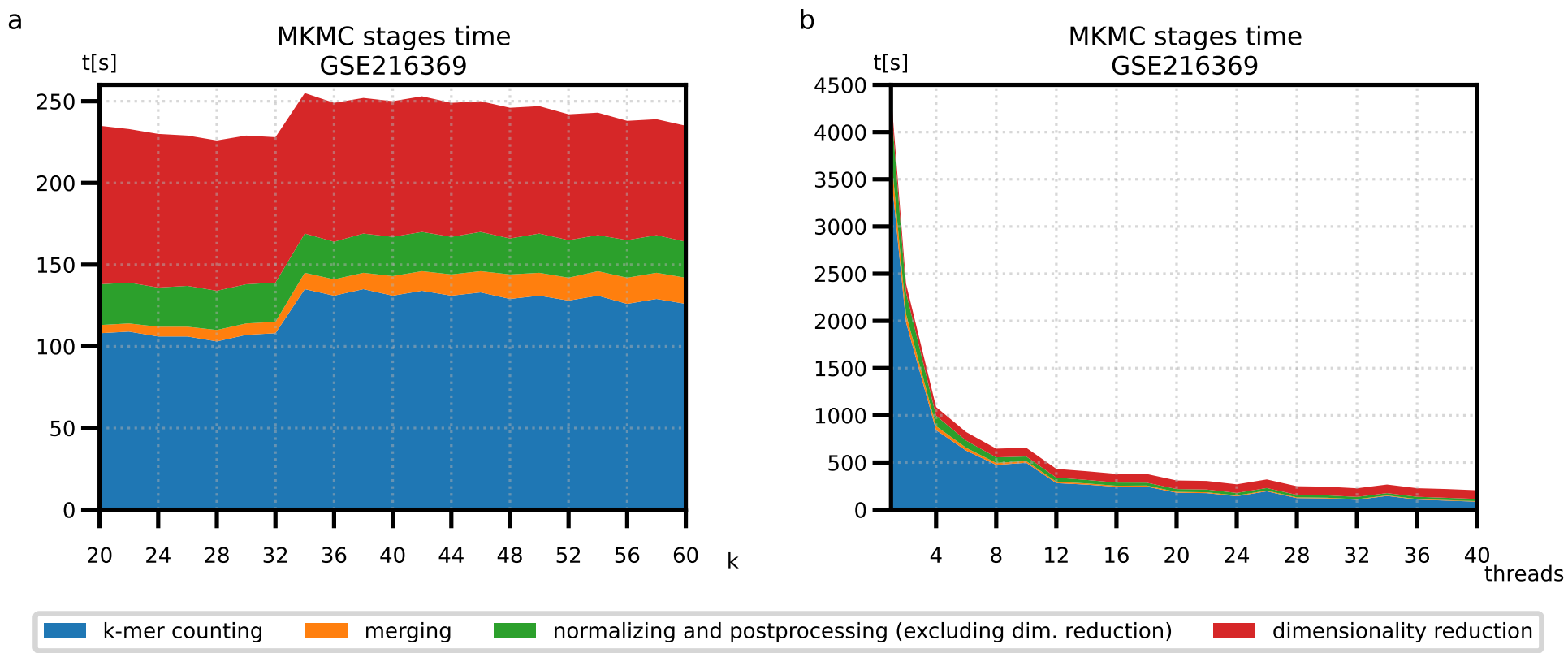

Fig. 9: Time of various MKMC stages depending on k-mer length and number of threads. MKMC was utilized to perform all the tasks in a single run: building the matrix and filtering, normalizing, and the matrix postprocessing. The plot shows time of its main stages (k-mers counting, merging — to build a matrix and filter) depending on a value of of k, and the number of threads.

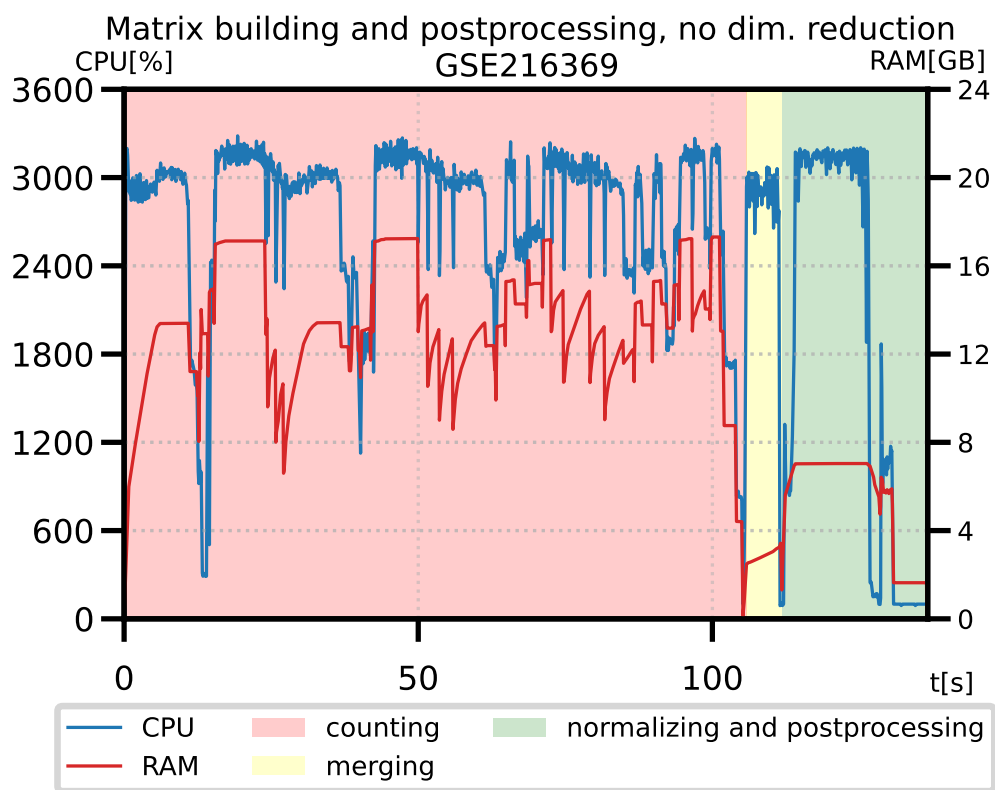

Fig. 10: CPU utilization (32 threads) and memory usage by MKMC performing all the tasks, except dimensionality reduction.

Table 5: Datasets utilized in experiments.

Dataset	Number of samples	Raw data size	Std. deviation of a single sample size	Number of reads
GSE216369	16	224.28 GB	1.613 GB	2.45G
GSE132040	933	12.49 TB	4.689 GB	177.2G

bioRxiv preprint doi: https://doi.org/10.64898/2026.07.06.736868; this version posted July 10, 2026. The copyright holder for this preprint (which was not certified by peer review) is the author/funder, who has granted bioRxiv a license to display the preprint in perpetuity. It is made available under a CC-BY 4.0 International license.

To measure wall-clock times and peak memory consumptions we utilized GNU time tool. CPU usage and memory consumption profiles (Fig. 3g and supplementary Fig. 9) were generated with Python cprof tool available in the GitHub repository. All the experiments were repeated once.

13.4.1 Command lines

Main time and memory benchmarks (subplots of Fig. 3 and, when stated, supplementary Fig. 9, 10) show results obtained by running MKMC, kmtricks, and Kamrat with the following command lines. MKMC and kmtricks generated binary matrices only. Kamrat indexed matrix in the text form, which was generated by merging with joinCounts results of Jellyfish dump.

MKMC — building a matrix (a, b, c, f, h)

```
mkmc -k <k> -m 16 --thr-rat 0.5 --thr 1 -t <threads> --
   reuse-db -- <samples_file> <output_name> tmp
```

The binary matrix (.kmcdb and .stats files) has not existed before the running. The running also collects data for frequency normalization.

MKMC — postprocessing counts by determining correlation (d, e)

```
mkmc -k <k> -m 16 --thr-rat 0.5 --thr 1 -t <threads> -n
   freq --cor <correlation_method> -p <
   correlation_phenotype> --reuse-db -- <samples_file> <
   output_name> tmp
```

The binary matrix has been generated before the running.

MKMC — postprocessing counts by differential k-mers analysis (d, e)

```
mkmc -k <k> -m 16 --thr-rat 0.5 --thr 1 -t <threads> -n
   freq --diff <de_method> -c <de_phenotype> --reuse-db --
    <samples_file> <output_name> tmp
```

The binary matrix has been generated before the running. Normalization (`-n freq`) was not used for T-Test.

Additionally, ANOVA, WRS, and T-Test were run also with p-values normalization with a parameter `--pval-corr b`.

MKMC — postprocessing counts by computing entropy (d, e)

```
mkmc -k <k> -m 16 --thr-rat 0.5 --thr 1 -t <threads> --
   entropy --reuse-db -- <samples_file> <output_name> tmp
```

The binary matrix has been generated before the running.

bioRxiv preprint doi: https://doi.org/10.64898/2026.07.06.736868; this version posted July 10, 2026. The copyright holder for this preprint (which was not certified by peer review) is the author/funder, who has granted bioRxiv a license to display the preprint in perpetuity. It is made available under a CC-BY 4.0 International license.

MKMC — building a matrix and performing various tasks in single run (g; supplementary Fig. 9)

```
mkmc -k <k> -m 16 --thr-rat 0.5 --thr 1 -t <threads> --flt
    <genome> -n freq --save-n --diff snr wrs dids anova
    ttest -c <de_phenotype> --pval-corr b --cor pearson
    spearman kendall -p <correlation_phenotype> --cv --
    leave 4 --entropy --umap --pca -- <samples_file> <
    output_name> tmp
```

MKMC — building a matrix and performing various tasks in single run, but no dimensionality reduction (supplementary Fig. 10)

```
mkmc -k <k> -m 16 --thr-rat 0.5 --thr 1 -t <threads> --flt
    <genome> -n freq --save-n --diff snr wrs dids anova
    ttest -c <de_phenotype> --pval-corr b --cor pearson
    spearman kendall -p <correlation_phenotype> --cv --
    leave 4 --entropy -- <samples_file> <output_name> tmp
```

The binary matrix has not existed before the running.

Where:

- `<k>` — k-mer length,
- `<threads>` — number of threads,
- `<correlation_method>` — one of `pearson`, `spearman`, `kendall`,
- `<correlation_phenotype>` — a file with phenotype/design,
- `<de_method>` — one of `anova`, `dids`, `snr`, `ttest`, `wrs`,
- `<de_phenotype>` — a file with phenotype/design for differential k-mers analysis,
- `<genome>` — genome file (GCF_001465895.1_Nfu_20140520_genomic.fna) in FASTA format,
- `<samples_file>` — a file with names of FASTQ files,
- `<output_file>` — output files names template.

kmtricks — building a matrix (a, b, c, f, h)

```
kmtricks pipeline --file <samples_file> --run-dir <dir> --
    kmer-size <k> --hard-min 1 --recurrence-min <
    recurrence_min> --mode kmer:count:bin -t <threads>
```

Where:

- `<k>` — k-mer length,
- `<threads>` — number of threads,
- `<recurrence_min>` — a half of the number of samples,
- `<samples_file>` — a file with names of FASTQ files,
- `<dir>` — an output directory.

bioRxiv preprint doi: https://doi.org/10.64898/2026.07.06.736868; this version posted July 10, 2026. The copyright holder for this preprint (which was not certified by peer review) is the author/funder, who has granted bioRxiv a license to display the preprint in perpetuity. It is made available under a CC-BY 4.0 International license.

Kamrat — building a matrix (a, b)

For every sample $i = 1, 2, \ldots, N$ Jellyfish was called:

```
jellyfish count -t <threads> -C -m <k> -s 300M -o <
   tmp_jf_db> <(zcat <fastq_file_1>) <(zcat <fastq_file_2
   >)
jellyfish dump -c <tmp_jf_db> | sort --parallel <threads> -
   S 16G -k 1 > <dump_file_i>
```

Then Jellyfish dumps were used to build a matrix in text form and run Kamrat:

```
joinCounts <dump_file_1> <dump_file_2> ... <dump_file_N> >>
    <text_matrix>
apptainer exec KaMRaT.sif kamrat index -intab <text_matrix>
    -outdir <dir> -klen <k> -unstrand -nfbase 1000000
```

Kamrat — scoring k-mers (the matrix postprocessing) with design (d)

```
apptainer exec KaMRaT.sif kamrat score -idxdir <dir> -
   scoreby <score_method> -design <design> -outpath <
   score_method>.csv
```

Kamrat — scoring k-mers (the matrix postprocessing) by computing entropy (d)

```
apptainer exec KaMRaT.sif kamrat score -idxdir <dir> -
   scoreby entropy -outpath entropy.csv
```

Where:

- `<k>` — k-mer length,
- `<threads>` — number of threads,
- `<fastq_file_1>`, `<fastq_file_2>` — paired input FASTQ files for single sample,
- `<tmp_jf_db>` — Jellyfish output database,
- `<dump_file_1>`, `<dump_file_2>`, `<dump_file_i>`, `<dump_file_N>` — joinCounts consecutive output files,
- `<text_matrix>` — the matrix in text format,
- `<dir>` — an output directory,
- `<score_method>` — one of `pearson`, `spearman`, `snr`, `ttest.padj` (enables also Benjamini-Hochberg correction), `dids`,
- `<design>` — a file with phenotype/design.
